# Exploring Law Enforcement Officers’ Experiences with Athletic Trainers and Work-Related Injury

**DOI:** 10.3390/ijerph22121769

**Published:** 2025-11-21

**Authors:** Stephanie Clines, Rubie Gaudette, Amanda Wheeler, Jennifer Ostrowski

**Affiliations:** 1 Department of Physical Therapy & Human Movement Science, Sacred Heart University, Fairfield, CT 06825, USA; 2 School of Rehabilitation Sciences, Moravian University, Bethlehem, PA 18018, USAostrowskij@moravian.edu (J.O.); 3 Department of Occupational Therapy, Sacred Heart University, Fairfield, CT 06825, USA; wheelera2@sacredheart.edu

**Keywords:** public safety, police, occupational health, musculoskeletal injury, tactical athletes, health services utilization, injury minimization

## Abstract

Law enforcement is an emerging clinical setting for athletic trainers (ATs), yet little is known regarding law enforcement officers (LEOs) perceptions of the profession. This qualitative study following a general inductive approach explored LEOs’ experiences with musculoskeletal injury and their interactions with ATs. Seven officers (*N* = 7) participated in one-on-one semi-structured interviews. Three themes emerged: (1) roles and responsibilities of ATs, (2) education and training, and (3) impact of injury on LEOs. While participants viewed ATs positively, they demonstrated limited understanding of the profession’s scope and expertise. Participants commonly described managing musculoskeletal injuries on their own due to cultural expectations to “push through” pain, limited organizational support, and administrative barriers to care. These patterns reflect broader occupational health and safety concerns within law enforcement and highlight opportunities for ATs to enhance injury prevention and early intervention by promoting a more supportive safety climate and improving access to musculoskeletal care.

## 1. Introduction

Law enforcement officers (LEOs) work in stressful, physically demanding, unpredictable, and dangerous environments [1], frequently involving rough terrain and diverse weather conditions. In addition to environmental challenges, the physical, technical, and equipment demands place LEOs at a heightened risk for musculoskeletal injury and postural imbalances [2]. Work-impacting and work-related injury rates for LEOs and Sheriff patrol officers are among the highest of all occupations [3,4], commonly consisting of musculoskeletal issues such as lower extremity sprains and strains and shoulder pathology [5]. Musculoskeletal injuries in first responders affect job performance, impact activities of daily living, result in workers compensation claims that add financial strain on the individual and state, and place burdens on the workforce of the organization [6,7,8,9]. Additionally, performance deficits resulting from musculoskeletal injury can place LEOs at a higher risk for harm and subsequent catastrophic consequences.

Despite awareness of the increased risk of musculoskeletal injury among LEOs, there remains a persistent need for evidence-based strategies in both prevention and management [10]. Members of the armed forces represent a parallel population, experiencing similarly high incidence of musculoskeletal injuries as well as the physical, personal, organizational, and national burdens associated with these occurrences [11]. In response, the U.S. military has integrated athletic trainers (ATs) into its health care teams to help better prepare and protect services members [12].

Athletic trainers are specialized health care providers with expertise in the assessment, diagnosis, prevention, treatment and rehabilitation of emergent and non-emergent injuries and illnesses in physically active populations [13]. Their versatile skill set allows them to serve across a range of clinical settings, including traditional athletics, performing arts, industrial and occupational health, physician offices, and the military [14]. The integration of ATs within the military has demonstrated a reduction in injury related attrition as well as positive financial outlook [15,16], with return on investment as high as US$ 9.00 for every US$ 1.00 spent on AT-led injury prevention programs [17].

While the military model demonstrates the potential value of embedded ATs, direct translation to law enforcement may be limited by differences in organizational structure, culture, and health care access. Military personnel typically operate within centralized systems that emphasize early reporting and mandatory injury care [17], whereas law enforcement officers often rely on decentralized or external medical resources [6], which may create barriers to timely care and rehabilitation. Moreover, organizational culture and safety climate differ between these populations, as military readiness initiatives aim to normalize early reporting [18], while law enforcement culture often emphasizes toughness and self-reliance, discouraging help-seeking for physical or psychological concerns [8,19]. These contextual differences may influence officers’ willingness to engage with ATs or other health professionals, underscoring the need to examine how organizational factors shape care-seeking behaviors in law enforcement.

Building on the success of the military model, public safety agencies, including law enforcement, are beginning to recognize the value of ATs in supporting injury prevention and care for their personnel [20]. Despite this emerging interest, the practice setting remains relatively new; only 6% of all National Athletic Trainers’ Association members currently work in non-traditional settings such as public safety, military, or occupational health [14]. However, limited research has examined how organizational culture, safety climate, and perceived stigma toward injury influence LEOs’ engagement with athletic training services. To our knowledge, no previous literature has specifically explored LEOs’ perceptions of or interactions with ATs. Therefore, this study draws on concepts of organizational culture, safety climate, and health-seeking behavior to explore how LEOs perceive and experience ATs in their work environment. The following research questions guided this investigation: (1) What are LEOs’ experiences with ATs? (2) What are LEOs’ perceptions of ATs? (3) How do LEOs describe their injury tolerance?

## 2. Methods

Due to the exploratory nature of this study, a qualitative methodology following the general inductive approach [21,22] was selected based on our main objective to understand LEOs’ experience with ATs and athletic training services. This approach aligns with a social constructivist paradigm, which assumes that meaning is co-constructed through participants’ lived experiences and the researchers’ interpretation of those experiences [22].

### 2.1. Participants

Participants were eligible for inclusion if they actively held the position or rank of police officer, patrol officer, police corporal, sergeant, lieutenant, captain, deputy police chief, sheriff, detective, or chief of police within a law enforcement department in the United States at the time of data collection. Participants were recruited through a combination of convenience, criterion, and snowball sampling. Initially, convenience and criterion sampling were used to identify eligible participants meeting the inclusion criteria. Snowball sampling was then employed to reach additional officers through participant referrals.

A total of seven LEOs (4 males, 3 females; age 35 years ± 9 years), representing multiple departments across the state of Florida and one department in Nevada participated (Table 1). Pseudonyms were used to protect the participant’s identities. This study sought depth of insight rather than breadth, and small samples are typical in exploratory qualitative research, where the goal is rich description rather than statistical generalization [23]. Therefore, data saturation guided participant recruitment and was determined when no new codes or themes emerged during ongoing analysis [22,24].

### 2.2. Instrumentation

A semi structured interview guide was developed by the researchers, informed by previous literature regarding injury risk in LEOs [2] and the perceptions of ATs working in similar settings [25]. The interview guide consisted of two main sections: (1) a demographic questionnaire and (2) open-ended questions regarding LEOs experience with athletic training services. Prior to data collection, the interview guide was pilot tested for clarity and fluidity of questioning with an individual representing the inclusion criteria. No changes to the interview guide were made as a result of pilot testing. Data from the pilot test was not included in the final analysis.

### 2.3. Data Collection Procedures

Institutional review board approval was obtained prior to recruitment and data collection. One-on-one in-depth interviews were conducted with participants via the web-based meeting platform, Zoom (Copyright ©2025 Zoom Video Communications, Inc., San Jose, CA, USA). This format was selected because it allowed participants to more richly describe and elaborate on their personal experiences with ATs in a comfortable and accessible setting [24,26]. To ensure consistency, all interviews were conducted by the same researcher and lasted approximately 30 min. With participants’ prior consent, only the audio portion of each interview was recorded using Zoom’s built-in recording feature. Audio recordings were transcribed verbatim by the same researcher. Participant recruitment and data analysis followed a constant comparison approach to guide iterative coding and determine when saturation had been reached [22].

### 2.4. Data Analysis

Data was analyzed using a manual coding process consistent with a general inductive approach to allow key themes to emerge naturally in relation to the study aims [21]. The analytic process followed the principles of constant comparative analysis to enhance the rigor and credibility of the findings. Two researchers independently reviewed each transcript to identify initial codes that reflected meaningful segments of text. Following independent review, the researchers met to discuss and compare their coding decisions, reconcile differences, and collaboratively refine a unified codebook. This iterative process ensured consistent application of the coding framework across all transcripts and strengthened inter-coder reliability. The constant comparison process continued throughout analysis, with codes and categories repeatedly compared within and across interviews to refine definitions and relationships until thematic saturation was reached [22,23].

To further enhance trustworthiness, multiple-analyst triangulation was employed through the inclusion of an independent peer reviewer. The peer reviewer possessed qualitative research expertise and relevant contextual experience with law enforcement populations. This individual reviewed the coding structure, category organization, and thematic framework to verify interpretive alignment with the data and to minimize potential researcher bias. Revisions were made collaboratively based on the peer reviewer’s feedback to ensure that final themes accurately represented participant perspectives and maintained analytic transparency [22].

To account for researcher positionality and reflexivity, the research team engaged in ongoing self-reflection throughout data collection and analysis. Both primary coders were certified ATs with clinical experience working with traditional athlete populations, but without prior clinical experience or personal connections to law enforcement populations. This outsider perspective helped reduce potential bias stemming from insider assumptions while still allowing the researchers to interpret findings through the lens of professional expertise in musculoskeletal injury and rehabilitation. Reflexive memos were maintained to document analytic decisions and evolving interpretations, and regular team discussions were used to critically examine assumptions and maintain transparency. Input from the independent peer reviewer further supported reflexivity by providing an external perspective on data interpretation and theme development [22].

## 3. Results

Three major themes were identified in the analysis. Two themes reflected LEOs perceptions of ATs: (1) roles and responsibilities and (2) education and training. The third theme, impact of injury to LEOs, captured participants’ personal experiences with injury and how these experiences shaped their views on injury management.

### 3.1. Roles and Responsibilities

Participants’ descriptions of the duties of ATs were interpreted through the lens of the five domains of practice outlined in the BOC Practice Analysis, 8th Edition [27]. These domains include (1) Risk Reduction, Wellness and Health Literacy, (2) Assessment, Evaluation and Diagnosis, (3) Critical Incident Management, (4) Therapeutic Intervention, (5) Healthcare Administration and Professional Responsibility. While the domains were not used to define subthemes, they provided a useful framework for organizing and interpreting participants’ perceptions of ATs’ roles.

Overall, LEOs demonstrated a general understanding of AT’s responsibilities, most commonly referencing tasks related to injury evaluation, prevention, rehabilitation, and physical fitness. Responses varied in complexity depending on participants’ prior exposure to athletic training services. Law enforcement officers’ with previous experience working with an AT, typically as former high school or college athletes, provided more detailed descriptions and cited specific examples spanning multiple domains of practice, including the use of therapeutic modalities, taping, wrapping, and bracing, and concussion evaluation. Gracie reflected on her experience observing ATs during her time as a student-athlete:

*Any time you had any pain or any injuries, they would sort of monitor, observe, and track what’s going on with your injury and try to see possible treatment routes if that’s like stretching, stim, icing, heating, that type of stuff… They would do concussion testing at my school. They would do all your taping, stim and cupping… A bunch of different stretches and different techniques to do when something is wrong.*

In contrast, LEO’s without previous experience with an AT often described the profession in more generalized or inaccurate terms, frequently referencing duties related to personal training or strength and conditioning. When asked to describe the role of an AT, Roger responded, “Someone who’s knowledgeable about how the body works…someone who knows the mechanics of how the body goes, periodization with training, and stuff like that.”

Table 2, developed using enumeration [28], presents the frequency with which participant responses aligned with each of the five domains for athletic training practice, providing additional context for how LEOs conceptualize the athletic training profession.

### 3.2. Education and Training

The education and training theme reflected participant’s perceptions of the qualifications to practice as an AT. Regardless of prior experience with an AT, most participants acknowledged that formal education and training were necessary. However, their understanding of these requirements was broad and generalized, often referencing college degrees, assorted certifications, clinical experience or field work, or some combination of these elements. Danny described his perception of the educational requirements for an AT:

*I would say it is someone who has been to some sort of accredited school or university that has some sort of degree and/or some sort of certificate that is comparable to a degree. So, I would think that you would have either a degree, certificate, and “x” amount of training hours in the field.*

Some participants did speculate the types of course work or content that would be included in the education of an AT, identifying sports performance, anatomy and physiology, biology, exercise science, and kinesiology as academic areas related to the athletic training profession.

### 3.3. Impact of Injury to LEOs

Participants described how injuries influence their ability to fulfill job responsibilities, with both formal organizational responses and informal personal adjustments shaping their work performance. Across responses, injury was positioned as a routine and often unavoidable part of the profession, influencing how they perceive their own capabilities, interact with their work environment, and navigate systems of care. Five subthemes were identified: (1) duty reassignment, (2) personal modifications, (3) injury management strategies, (4) systematic barriers to care, and (5) job-related injury risk.

#### 3.3.1. Duty Reassignment

Participants commonly associated injury with formal restrictions on their ability to carry out standard active-duty responsibilities. In many cases, being injured meant reassignment to modified or light duty, often with limited physical requirements. When asked to define what it means to be injured Olivia explained, “to be injured means we can’t be on duty. We would be sitting at the desk or at some other civilian department because we can’t go about our normal duties.” Danny described a similar experience, framing injury as a spectrum that directly determines whether an officer is allowed to function in their full role:

*For me personally, an injury would mean anything that would preclude me from being able to function on a daily capacity at work. Whether that means light duty where I may be placed at the front desk for a certain injury, or more of a significant injury where I would need to stay home and I couldn’t carry a gun and a badge. It’s a sliding scale of injured; there’s the lower end where you could be placed at the front desk for desk duty or the higher end of that spectrum where they would take your gun and your badge and you wouldn’t have the ability to function as a police officer.*

#### 3.3.2. Personal Modifications

In addition to formal job restrictions, participants described making personal decisions to modify their approach to daily tasks when experiencing pain or injury, particularly in situations that require heightened physical engagement. Such self-regulation reflected not only a concern for personal safety, but also a perceived responsibility to minimize risk to others during physically demanding encounters. Roger shared how discomfort or vulnerability influenced his tactical decisions on patrol:

*Instead of doing proactive work like traffic stops, looking for warrants, I might be more prone to responding to calls for service because if I have a nagging injury—my two biggest injuries are my right shoulder and lower back—if either of them are aggravated or bothering me I’m going to be more cautious when it comes to getting in a situation where I need to fight someone or chase someone… I’m going to be more apprehensive because I know I’m more of a liability.*

#### 3.3.3. Injury Management Strategies

A strong culture of self-reliance emerged in how participants discussed managing injuries. Rather than seeking formal care, many officers reported using self-assessment techniques and informal treatment strategies to determine whether an injury required medical attention. Danny noted, “a lot of [LEOs] suffer minor injuries and keep that to themselves because they can work through them.” Roger detailed his process of evaluating injuries independently:

*My current process is primarily self-diagnosis. I’ll just self-determine if something hurts bad enough. I’ll check different ranges of motion, different movements, see if it’s a breakdown in form or if it’s actually aggravating a muscle. Whether it be an injury to the muscle like a strain or overuse, or if it’s a nagging injury I have had for a long period of time I just kind of go through that [assessment], but I don’t go get diagnosed for anything.*

The preference of self-management was also discussed in the context of injury severity, where LEO’s felt they were responsible for managing routine aches and pains that did not require significant medical care. When asked whether having a healthcare provider, such as an AT, available would be beneficial, Elliot contextualized his answer through a lens of enduring chronic pain:

*Police officers are regularly suffering injuries and don’t go seek treatment for them. I’m 25 years old and have severe lumbar pain from sitting in my car with my gun belt on all day. It would be amazing if a police department had someone on staff to help with things like that.*

#### 3.3.4. Systematic Barriers to Care

For more serious injuries requiring disclosure, participants expressed frustration with the formal Workers’ Compensation system. These frustrations centered on the lack of autonomy in choosing providers and the bureaucratic nature of care coordination. Jane shared her experience managing a significant work-related injury:

*You have to go to the [designated] trauma center [first]… [Then] you go to a specialist that workman’s comp is going to recommend and unfortunately for us our workman’s comp is not the easiest to deal with, which is kind of what I hear from many other departments as well. So [the worker’s compensation agency] picks your specialist, they pick your surgeon, and they pick your rehab. And again, you just go through the motions in order to make sure you get yourself back to full duty.*

Although the formal system provides a pathway to recovery, participants described it as inflexible and inefficient, contributing to a sense of disempowerment during the healing process.

#### 3.3.5. Job-Related Injury Risk

Participants viewed injury as an expected and accepted part of the profession. The physical nature of law enforcement, combined with the use of equipment and unpredictable field encounters, made discomfort, pain, and even serious injuries commonplace. Participants shared experiencing a wide range of work-related injuries including meniscal tears, dog bites, gunshot wounds, shoulder pathologies, fractures, chronic low back pain, neck pain, sprains, strains, contusions, and lacerations. Olivia described her experiences with injuries:

*We get injured a lot on the job. Whether you get in a foot chase and you’re chasing after somebody, you may fall in a hole, or you may climb over a fence to chase somebody and cut your hand open. Or even going physically with somebody, say you have to go hands on with somebody; I’ve seen [officers] get punched in the face, thrown to the ground. There’s really all kinds of injuries; dog bites, shootings… We are prone to get injured. We have a very physical job.*

The physical burden of wearing standard gear was also cited as a persistent source of strain. Additionally, some participants reported operating unique or large equipment. Jane’s duties, for example, include patrolling on a motorcycle:

*I ride a motorcycle for a living so I would say most everyone I work with [in the motorcycle unit] absolutely has back aches and pains; legs, knees, shoulders, just from riding the bike that you just deal with. That’s not something you are going to get checked out, you’re just going to do it.*

## 4. Discussion

Knowledge regarding the roles and responsibilities of ATs was directly influenced by participant’s previous experience receiving or observing athletic training services. Consistent with previous research among athletic directors [29,30], parents [31,32], legislators [33], and other healthcare providers [25], LEOs who had previous experience with an AT, particularly as former athletes, demonstrated a stronger understanding of the profession than those without such experience. Their descriptions, however, were largely confined to three of the five domains of athletic training practice [27], indicating an incomplete understanding of the full scope of the profession. In contrast, participants without prior exposure to ATs often conflated athletic training with personal training, focusing primarily on fitness, wellness, and nutrition. While these areas overlap with aspects of Domain 1 of the BOC Practice Analysis [27], they reflect a limited understanding of the AT’s broader role within healthcare. This misunderstanding underscores the persistent gap in public awareness regarding the distinct competencies that differentiate ATs from other allied health and fitness professionals.

Debate over the title “athletic trainer” has persisted for decades [34,35], largely due to concerns that it does not adequately reflect the profession’s full scope of practice. While the NATA’s Nomenclature Task Force concluded that a title change would offer limited benefit [36], the ongoing discussion highlights a broader issue: the general public, and particularly individuals outside of athletics, lack a comprehensive understanding of the AT’s expertise. Research has shown that direct exposure to ATs improves understanding [29], while those without such experience hold narrower or inaccurate views [33]. Relying solely on personal experience to shape understanding leaves room for misinterpretation. As the profession continues to expand into emerging settings, including law enforcement, advocacy and education must remain central to professional efforts to ensure stakeholders recognize the value ATs bring to physically demanding and high-risk occupations [37,38].

Despite variability in understanding the athletic training profession, participants consistently expressed interest in having access to ATs or similar providers within their departments. They recognized the potential value of ATs in managing chronic aches and pains commonly accepted as part of the job. Many participants described ongoing musculoskeletal discomfort associated with duty gear, patrol work, and job-related strain, viewing it as an inevitable aspect of policing. The appeal of on-site or embedded support was tied to convenience, trust, and accessibility, factors previously identified as key to successful integration of healthcare providers in military and tactical settings. In these populations, embedded providers, including ATs, have improved injury reporting, shortened treatment delays, and enhanced readiness through early intervention and prevention [39]. Given the parallels between law enforcement and military work, it is reasonable to expect similar benefits if ATs were integrated into police departments [15,16]. Future research should examine these outcomes quantitatively, assessing impacts on injury incidence, recovery time, and operational readiness, as well as potential organizational cost savings

Cultural and institutional barriers, however, remain significant challenges to effective musculoskeletal care. Participants described a prevailing expectation to “push through” pain, minimizing injury unless it was severe enough to disrupt job performance. This mindset provides insight into the safety climate within law enforcement. A weak safety climate, characterized by limited organizational support for reporting and addressing injuries, can discourage early intervention and contribute to adverse health outcomes [40,41]. Likewise, presenteeism, or working despite injury or illness, has been associated with decreased productivity, prolonged recovery, and increased risk of reinjury [42,43]. Officers also cited logistical challenges, including navigating Workers’ Compensation systems, taking personal time for appointments, and lack of control over provider choice, all of which disincentivized care-seeking. These findings mirror patterns seen in the military [39], where fear of being sidelined, stigma, or frustration with the formal reporting process often delayed care. Addressing these intertwined cultural and procedural barriers requires both organizational and educational strategies that promote early reporting, reduce stigma, and emphasize health as integral to readiness and safety.

Injury minimization, particularly among this population, is concerning given its implications for performance and long-term impacts. Delayed care increases the likelihood of secondary or chronic injury and contributes to greater healthcare utilization and costs [44,45,46,47]. Musculoskeletal pain has been shown to directly influence job readiness in physically demanding occupations [12,44,48], compromising not only individual safety but also that of colleagues and the public.

Importantly, participants highlighted a cultural phenomenon that differentiated between pain and injury in ways that may influence care-seeking behavior. While athletes describe injury on a sliding scale of severity influencing performance at varying levels [49], LEOs tended to view injury as a binary state, holding an “all or nothing” perspective on what it means to be injured, e.g., either severe enough to remove them from duty or not an “injury” at all. Pain, by contrast, was viewed as an expected occupational experience, part of the physical toll of policing. This distinction reflects the influence of professional identity and cultural norms that prioritize operational performance over self-care [50]. Educational initiatives that help officers conceptualize pain as a potential indicator of injury rather than a normal part of the job could facilitate earlier intervention and improve long-term outcomes.

Integrating ATs within law enforcement departments may help address both the cultural and structural barriers identified in this study. Athletic trainers are uniquely positioned to support LEOs because of their expertise in orthopedic injury management, prevention, and rehabilitation among physically active populations. Their familiarity with environments emphasizing toughness and performance [51,52], along with specialized knowledge regarding personal protective equipment and task-specific demands [30,53], make them particularly well-suited for this role [54]. Embedding ATs could help normalize early reporting, reinforce a positive safety climate, and foster a culture in which seeking care is viewed as an operational strength rather than a weakness. As licensed healthcare providers, ATs are guided by professional standards and ethical principles that prioritize patient-centered care under the supervision of a physician [13]. Maintaining clear communication channels and well-defined roles within the department can further ensure alignment between individual care and organizational goals, supporting both officer well-being and operational readiness.

While participants were recruited from multiple agencies, most were employed within a single state, limiting geographic diversity in perspectives. Additionally, none had direct experience receiving care from an AT while serving as a LEO. Despite these limitations, the study offers valuable preliminary insight into perceptions of athletic training, barriers to care-seeking, and opportunities for interprofessional collaboration to enhance the health and safety of the law enforcement workforce.

## 5. Conclusions

This study provides new insight into how law enforcement officers perceive and navigate musculoskeletal injury within a culture that prioritizes performance and resilience. Findings highlight how structural and cultural barriers, such as limited organizational support for injury reporting, stigma around help-seeking, and complex administrative processes, delay care and contribute to prolonged recovery. Athletic trainers are uniquely equipped to mitigate these challenges by providing early intervention, rehabilitation expertise, and education that align with the operational demands of policing.

By identifying both the opportunities and perceived barriers to integrating ATs in law enforcement, this study contributes to a growing understanding of how allied health professionals can enhance occupational health systems in high-risk populations. Future research should build on these findings by evaluating the impact of athletic training services on officer health, readiness, and organizational safety culture within public safety agencies.

## Figures and Tables

**Table 1:** Participant Demographics.

Pseudonym	Sex	Age	Years of Experience	Position Title	Department Location	Previous Experience with an AT
Roger	Male	27	2.5	Patrol Officer	Florida	No
Lennie	Male	47	20	Deputy Sheriff, SWAT, Patrol	Florida	Yes
Olivia	Female	32	5.5	Detective, Sergeant	Florida	No
Danny	Male	42	12	Detective	Florida	No
Jane	Female	44	13	Police Officer First class	Nevada	Yes
Elliot	Male	25	3	Police Officer, Field training	Florida	Yes
Gracie	Female	26	4	Police Officer First Class	Florida	Yes

Abbreviations: AT = Athletic trainer.

**Table 2:** Participant Cases by Theme (*N* = 7).

Theme and Subtheme	Frequency	No. of Cases
**Roles and Responsibilities**		
Risk reduction, wellness and health literacy	General	7
Assessment, evaluation and diagnosis	Typical	4
Critical incident management	Rare	1
Therapeutic intervention	Typical	4
Healthcare administration and professional responsibility	Rare	0
**Education and Training**	General	6
**Impact of Injury to Leos**		
Duty reassignment	General	6
Personal modifications	Variant	3
Injury management strategies	Typical	5
Systematic barriers to care	Typical	4
Job-related injury risk.	General	7

Frequency Components: General = all or all but one; Typical = 4 or more; Variant = 3 or less; Rare = only 1 case.

## Data Availability

The data supporting the findings of this study consists of qualitative interview transcripts that contain confidential and potentially identifiable information. As such, they are not publicly available in order to protect participant privacy. Relevant excerpts of the data are presented within the manuscript in accordance with qualitative data reporting standards.

## Abbreviations

The following abbreviations are used in this manuscript:

AT	Athletic trainer
BOC	Board of Certification, Inc.
LEO	Law enforcement officer
NATA	National Athletic Trainers’ Association
